# Acute Pancreatitis in COVID-19-associated Multisystem Inflammatory Syndrome of Children—A Single Center Experience

**DOI:** 10.1097/PG9.0000000000000150

**Published:** 2021-12-10

**Authors:** Bhaswati C Acharyya, Monideepa Dutta, Saumen Meur, Dhritabrata Das, Saumyabrata Acharyya

**Affiliations:** From the * Department of Paediatric Gastroenterology, AMRI Hospitals, Mukundapur, India; † Department of Paediatric Intensive Care, AMRI Hospitals, Mukundapur, India; ‡ Department of Paediatric Cardiology, AMRI Hospitals, Mukundapur, India; § Department of Paediatrics, AMRI Hospitals, Mukundapur, India.

**Keywords:** SARS-CoV-2, COVID-19, multisystem inflammatory syndrome in children, pancreatitis

## Abstract

A multisystem inflammatory syndrome in children (MIS-C) was identified as an entity temporally associated with the present COVID-19 pandemic. This inflammatory syndrome affects various organ systems including the gastrointestinal and hepatobiliary systems. The following study was undertaken to primarily detect the fraction of children who had pancreatitis as major organ involvement during the development of MIS-C. The secondary objective was to evaluate their clinical and investigational profile as well as the outcome of management.

**Methods:**

A retrospective chart analysis of all children admitted in a tertiary pediatric center from April to December 2020, diagnosed as COVID-19 associated MIS-C, was done. Those presenting with acute pancreatitis were detected and detailed clinical features, investigations, treatments, as well as outcomes, were recorded.

**Results:**

A total of 17 children were diagnosed as MIS-C associated with SARS-CoV-2 in the above-mentioned period. Among them, 9 (53%) children had pancreatitis right from the start of the illness; 5 (56%) children with pancreatitis presented with shock. A very high CRP and cardiac involvement were observed in all. A severe reduction of myocardial contractility was associated with poor outcomes. Necrotic pancreatitis was not noted in any of them. A mortality of 22% was documented in this group and a 3-month outcome among those who survived displayed complete resolution of all cardiac as well as pancreatic abnormalities.

**Conclusion:**

Acute pancreatitis can be an essential cause of abdominal symptoms in COVID-19-related MIS-C. Pancreatitis may be considered to be one of the parameters in the diagnostic criteria of MIS-C.

## Abstract 2

Supplemental Digital Content is available in the text.

**What Is Already Known**

- Multisystem inflammatory syndrome in children (MIS-C) is a known inflammatory disease presently identified associated with the COVID-19 pandemic.
- Acute pancreatitis (necrotic or nonnecrotic) can be seen in COVID-19 infection due to various pathophysiologic reasons.

**What This Study Adds**

- Acute pancreatitis can be a cardinal reason for abdominal pain in COVID-19-related MIS-C.
- Considering the morbidity, independent inclusion of pancreatitis might be considered in the diagnostic criteria of MIS-C.

## INTRODUCTION

In the initial days of the SARS-CoV-2 pandemic, it was believed that Coronavirus disease 19 (COVID-19) rarely causes very severe disease in the pediatric population (1). In April 2020, reports from the United Kingdom documented a presentation in children similar to incomplete Kawasaki disease (KD) or toxic shock syndrome (2). Since then, there have been reports of similarly affected children from various parts of the world (3,4). This entity was named multisystem inflammatory syndrome in children (MIS-C) temporally associated with COVID-19 (5). The first case from India was reported in April 2020 (6). Our group reported the first infant with Kawasaki-like manifestation in May 2020 (7). Since then, paediatricians in our unit became vigilant to diagnose COVID-19-related MIS-C early to manage these children efficiently with the help of the pediatric intensive care team. A prospective collection of data was started to keep the record of all those children with fever with or without shock for future research. Few of them had abdominal pain as the chief symptom along with fever which eventually evolved as pancreatitis associated with COVID-19-related MIS-C. So this retrospective analysis of the prospectively collected data was undertaken to detect the fraction of children with MIS-C presenting as acute pancreatitis. The objectives of this study were as follows:

(1) To find out the fraction of children diagnosed as acute pancreatitis in presence of MIS-C
(2) To evaluate the clinical and investigational profile of these children
(3) To evaluate the outcome of management of these children

## MATERIALS AND METHODS

### Study Setting

This study was undertaken in the pediatric intensive care unit and pediatric ward of a tertiary pediatric hospital in Eastern India.

### Study Period

April 2020 to December 2020

### Study Design

A retrospective observational study

### Study Population

Children with age 1 month to 18 years admitted as an inpatient with fever from April 2020 to December 2020 were included.

### Inclusion Criteria

Patients found to have MIS-C.

### Exclusion Criteria

(1) Children not having fever due to SARS-CoV-2 (by identifying RTPCR or COVID 19 antibody test or contact with active COVID-19 infected patients within a month)
(2) Children not diagnosed as COVID 19 associated MIS-C

### Procedures

**FIGURE 1.**
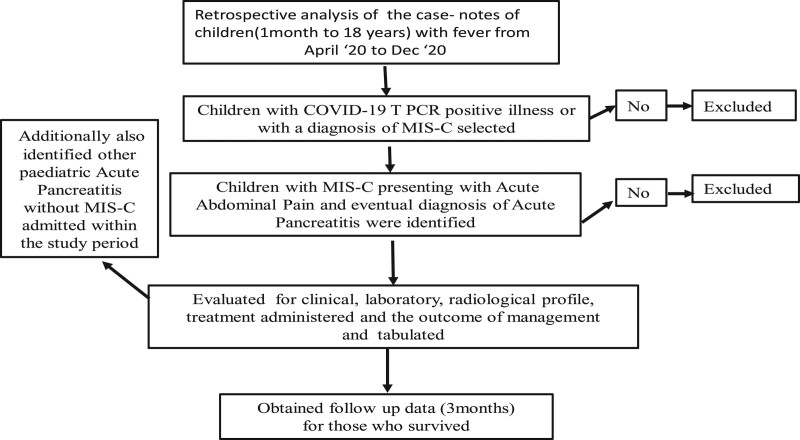
Flow chart of study design.

A retrospective analysis of case notes of all the children admitted with fever was undertaken (Fig. 1). Those who had COVID-19 RT-PCR positive results or had a diagnosis of MIS-C (though RT-PCR negative) were identified from these notes. Diagnosis of MIS-C was undertaken as per RCPCH description (5). Children presenting with abdominal pain with an eventual diagnosis of acute pancreatitis were identified to evaluate their clinical, laboratory, radiological profile, treatment administered, and the outcome of management. All these children had been investigated with complete blood count (CBC), C-reactive protein (CRP), liver function test (LFT), coagulation studies, D-dimer, amylase and lipase, blood and urine cultures, chest radiograph, and echocardiography. Patients with raised lipase, after initial abdominal ultrasound scan, had magnetic resonance cholangio pancreatography (MRCP) or CT scan of the abdomen as a pancreatic imaging study. Troponin C levels and interleukin 6 were estimated whenever necessary. Acute pancreatitis was diagnosed by Atlanta Criteria (8). Children with MIS-C who had negative COVID RT-PCR were tested for corona viral antibody. Treatment was supportive along with IV gammaglobulin at a dose of 2 g/kg infused over 2 days. Intravenous methyl-prednisolone was administered at a dose of 10–30 mg/kg when they presented with shock. Children with raised D dimers were administered low molecular-weight heparin (LMH) subcutaneous injection until the D dimer was normalized. Initially, intravenous antibiotics were administered until all cultures were negative. Children with AP were initially (first 24 hours) administered Ringer Lactate solution as per the hydration status, presence or absence of shock, and cardiac compromise with meticulous monitoring of intake and output. Injectable proton pump inhibitor (PPI), Esomeprazole was administered in two divided doses daily. Enteral feeding was started within 48 hours in all children; initially with clear liquid in the form of oral rehydration solution (ORS) and clear soup followed by all forms of liquid diet. Nasogastric feeding was practiced for those who could not take oral feeding initially. A soft diet was started as per tolerance. All children who recovered were followed up for 3 months in outpatients. During follow up, abdominal ultrasound and echocardiography were repeated in the first month and the third month. CBC, CRP, LFT, and serum lipase were rechecked in the first outpatient visit. D-dimer was repeated 1 week after discharge, for those who were discharged with low molecular-weight heparin (LMH) injection

### Ethical Approval

Ethical Committee approval was obtained from the Institutional Ethics Committee.

### Statistical Analysis

Simple statistical calculation was done using basic statistical methods with SPSS software for windows version 23.0 (SPSS, Chicago, IL). Descriptive statistics for categorical variables are described as counts and percentages. Nonnormally distributed continuous variables are described as median and interquartile ranges.

## RESULTS

A total of 1019 children were admitted during the above-mentioned period. Twenty-nine had COVID RT-PCR positive illness. Seventeen children had MIS-C (only 2 had RTPCR positive status). Nine children (53% of total MIS-C patients) presented with acute pancreatitis (AP). During this study period, none with acute SARS-CoV-2 infection without MIS-C had acute pancreatitis. Five other children were admitted to pediatric unit during this study period. None had corona viral infection. Two of them had recurrent acute pancreatitis due to SPINK and CFTR mutation, respectively, the third child had obstructed choledochal cyst with biliary pancreatitis, the fourth child was found to have the long common channel and the last had sodium valproate induced acute pancreatitis.

### Acute Pancreatitis Group of the MIS-C Subset

#### Demography

The median age of children was 10 years (IQR 6–13.5). The male and female ratio was 3:2. None of them had any comorbidity except one child (no. 1 in Table 1) had a BMI of more than 97th centile (Table 1).

**TABLE 1.** Demography and clinical features

Patient parameters	Child 1	Child 2	Child 3	Child 4	Child 5	Child 6	Child 7	Child 8	Child 9
Age (years)	13	9	7	14	10	15	4	5	11
Sex	F	F	M	M	M	F	M	M	M
vomiting	Yes	Yes	Yes	Yes	Yes	Yes	No	Yes	Yes
Reduced urine output	Yes	No	Yes	Yes	No	No	No	No	No
Fever and onset of pain (days)	2	2	1	2	3	1	1	2	2
Duration of illness before hospitalization (days)	7	4	3	12	6	4	5	6	3
Shock at presentation	Yes	No	No	Yes	Yes	Yes	No	No	No
Jaundice	Yes	No	No	No	No	No	No	No	No
Ascites	Yes	No	Yes	Yes	yes	Yes	Yes	No	No
Edema	Yes	No	Yes	Yes	Yes	No	No	No	No
Hepatomegaly	Yes	No	Yes	Yes	No	No	Yes	No	No

#### Clinical Profiles

The clinical profile of the patients are described in Table 1.

The median time to develop abdominal pain from the day of commencement of fever was 2 days (IQR 1–2). Shock was present in 5 (55.5%) children at the presentation. None had jaundice. Edema and ascites were noted in 4 (44%) and 6 (67%), respectively, at the presentation.

#### Investigations

Very high C reactive protein (CRP) was found in all of them (Table 2). Median CRP was 112 mg/L (IQR 89.3–181.05). Deranged liver functions (enzymes > 2 times of the upper limit of normal) were found in 5 (55.5%). The median albumin level was low at 2.6 g/dL (IQR 2.45–2.9). Raised D-dimer was found in 5 (55.5%).

**TABLE 2.** Investigations, treatments, and outcome of children with pancreatitis

Patient Parameters (normal upto)	Child 1	Child 2	Child 3	Child 4	Child 5	Child 6	Child 7	Child 8	Child 9
CRP (9 mg/L)	290	112.1	78.8	80.6	198.1	164	132	98	102
Lipase (150 U/L)	>6000	786	642	1863	2348	2600	862	466	2344
SGOT (55 U/L)	283	88	47	40	42	156	234	187	52
SGPT (34 U/L)	117	124	39	44	31	134	180	150	48
Albumin 3.8 g/dL (lower limit)	1.9	2.6	2.5	2.9	2.6	2.9	2.4	2.9	2.7
Ptime (15 s)	20.2	13.5	23	48.5	15.8	30.4	13.2		12.1
APTT (35 s)	36.9	30.5	40.9	37.7	33.7	50.4			
D-dimer (0.5 µg/mL)	5.6	1	9	14.5	2.67	3.4	3.2	1	1
USG abdomen	Hepatomegal, ascites, pancreas not well visualized	Mild ascites, hepatomegaly, heterogrneous pancreas	Bulky pancreas	Ascites, marked hepatomegaly, pancreas bulky	Mild ascites, enlarged spleen, bulky pancreas	Hepatomegaly, ascites noted, pancreas not well visualized	Hepatomegaly, perpancreatic fat stranding	Mild hepatomegaly, pancreas bulky with fluid around tail	Bulky pancreas
MRCP/CT abdomen	Could not be done	Bulky pancreas, marked fat stranding around head, body, and tail with peripancreatic collection, no necrosis, ascites noted (Fig. 2A)	Marked fat stranding around head with small collection of fluid around head	Could not be done	Very bulky pancreas, ascites seen	Bulky pancreas, fat stranding around the body, ascites seen	Bulky pancreas, fat stranding around tail, no ductal anomaly (Fig. 3A)	Marked fat stranding at body and tail with peripancreatic fluid collection, ducts normal, GB normal	Bulky pancreas, ducts and GB, and CBD normal, mild ascites
Echocardiography	Marked LV systolic dysfunction	Dilated LAD. Perivascular brightness and lack of tapering in LAD (Fig. 2B)	Pervascular brightness, with multiple soft signs of KD	Severe LV systolic dysfunction with generalized LV wall hypokinesia	Moderate LV systolic dysfunction, aneurysm in LAD. Dilated coronaries	Perivascular brightness with prominent coronaries	Dilated LMCA and LAD. Perivascular brightness and lack of tapering in LAD (Fig. 3B)	Coronary arterial ectasia, mild pericardial effusion	Mild LV, dysfunction, perivascular brightness, and lack of tapering in LAD
Treatment	Ventilation, vasoactive agents, steroid	IV gamma, aspirin	IV gamma, IV steroid, aspirin, LMH	Ventilation, IV gamma, IV steroid, vasoactive agents, aspirin, LMH	IV gamma, IV steroid, LMH	IV gamma, IV steroid, aspirin, LMH	IV gamma, aspirin, LMH	IV gamma, aspirin	IV gamma, aspirin
COVID-19 RTPCR	Positive						Positive		
Spike-protein antibody >0.8 U/ml -Pos		Positive Titer NA	69.2	4.5	Negative	Positive Titer NA		Positive Titer NA	Positive Titer NA
Hospital stay (days)	1	9	12	2	11	13	8	8	9
Outcome	Expired	Recovered	Recovered	Expired	Recovered	Recovered	Recovered	Recovered	Recovered

CBD = common b`ile duct; CRP = C-reactive protein; GB = gall bladder; IV gamma = intravenous gamma globulin; KD = Kawasaki Disease; LAD = left anterior descending artery; LMCA = left main coronary artery; LMH = low molecular weight heparin; LV = left ventricle; MRCP = magnetic resonance cholangio pancreatography; RCA = right coronary artery.

Cardiac involvement was detected in all the children with pancreatitis. Severe impairment of myocardial contractility was found in 2 (22.2%) children. None was found to have necrotic pancreatitis. Two cases were found to be COVID-19 RT-PCR positive, 6 others had corona viral antibody (IgG against spike protein) except one who did not have such documentary evidence of COVID-19 infection but had a positive family history of SARS-CoV-2 infection about 4 weeks ago.

#### Treatment

Intensive care admission was needed in 5 (55.5%). Intravenous gamma globulin at a dose of 2 g/kg was administered in all except one, who had a refractory shock and succumbed before the gamma globulin infusion could be initiated. Two children (22%) needed mechanical ventilation who did not survive eventually. Fever subsided within 48 hours of completion of gamma globulin in all survivors. Intravenous methyl-prednisolone was needed in 5 (55.5%) cases. LMH was administered in 55.5% of children.

#### Outcome

Seven children recovered well and 2 expired (22% mortality). Surprisingly in the whole MIS-C group, these 2 had the negative outcome. Two deaths in this series resulted in a mortality of 12% among the total MIS-C cases and 22% among the MIS-C with pancreatitis group. The use of methyl-prednisolone did not adversely worsen the course of pancreatitis in any of these patients. Follow-up of all 7 children could be completed up to 3 months. Complete recovery of cardiac abnormalities, as well as pancreatitis without any complication, was noted.

## DISCUSSION

Acute pancreatitis as a presentation of COVID-19 and that too in children with MIS-C has not commonly been described in the literature. This is the first series to show 53% of children of MIS-C associated with COVID-19, presenting as acute pancreatitis.

Although the initial description of MIS-C abdominal symptoms in the form of pain was described by Riphagen et al at the early phase of the pandemic pancreatitis was not specifically described or identified as the cause of the pain (2). In the description of MIS-C in the RCPCH position, paper pancreatitis was not mentioned in the list of organ involvement though abdominal symptoms were described (5). A recently published series by Feldstein et al, where characteristics and outcomes of Acute COVID-19 in children were compared with that of MIS-C, mentioned acute pancreatitis as an entity in 3% of children with MIS-C (in the supplementary content only http://links.lww.com/PG9/A62) (9). Abdominal pain was detected to be a manifestation in a mean of 70% of children in this meta-analysis by Yosuhara et al but they did not mention acute pancreatitis as the cause of the pain (10). Our finding of acute pancreatitis in 53% of children of MIS-C is a new and unique addition to the literature.

Adult data commenced stating pancreatic injury with COVID-19 infection from the first quarter of 2020 even before World Health organization declared it as a pandemic. Wang et al in their series of corona virus-19 pneumonia from china described 17% of cases with pancreatic injury. In their report, adults with pancreatitis had a more serious illness from the onset and 7 (out of 9) of them received steroids (11). This was before the identification of the multisystem inflammation associated with COVID-19. Since then, case reports and series with acute pancreatitis in COVID-19 infected adults had been reported from different countries (12–14).

**FIGURE 2.**
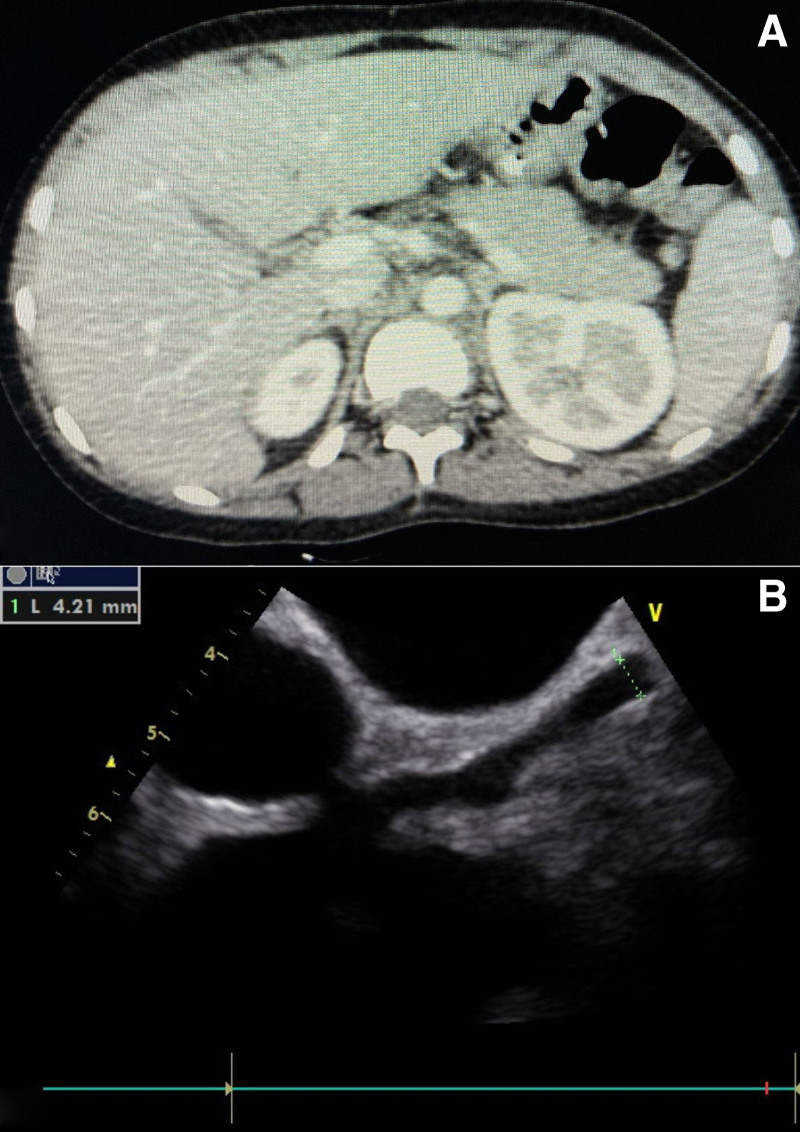
A) CT scan showing bulky pancreas, marked fat stranding around head, body and tail with peripancreatic collection. B) Echocardiography showing dilated LAD and lack of tapering in LAD. LAD = left anterior descending artery.

**FIGURE 3.**
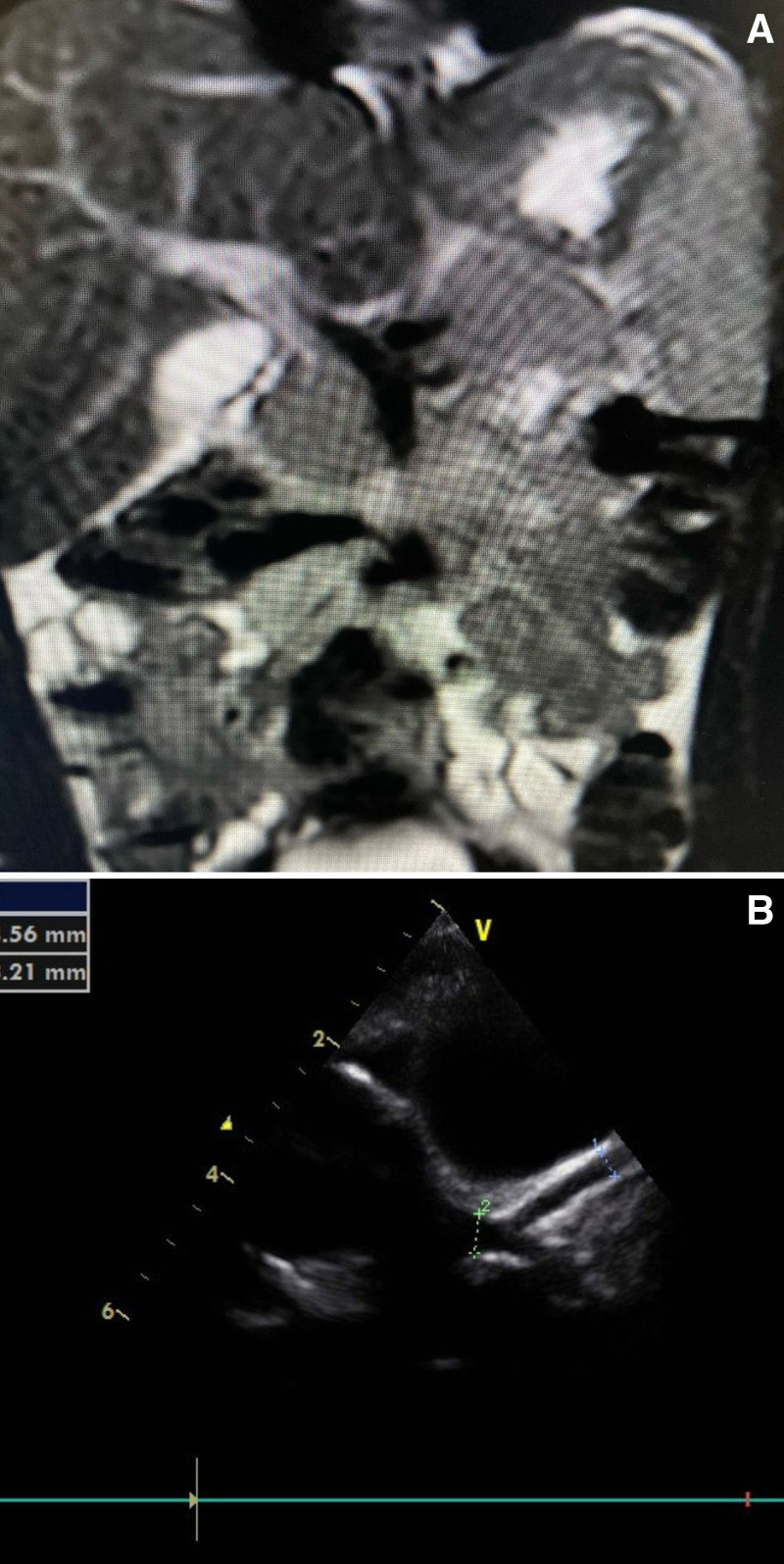
A) MRCP showing bulky pancreas and fat stranding around tail. B) Echocardiography showing dilated LMCA and LAD with perivascular brightness. LAD = left anterior descending artery; LMCA = left main colonary artery; MRCP = magnetic resonance cholangio pancreatography.

Pediatric case report of acute pancreatitis associated with COVID-19 disease was first seen in the literature in May 2020 by Alloway et al. They described a febrile 7-year-old girl presenting with abdominal pain in the emergency, incidentally found to be COVID-19, RTPCR positive (15). Subsequently, in July 2020, Samchez et al depicted a 16-year-old obese Hispanic child who had bibasilar pneumonia and AP as a manifestation in SARS-CoV-2 infection (16). A series of three children was illustrated in the report by Samies et al on October 2020 (17). In December, Suchman et al mentioned a 1.8% covid-19 positivity in hospitalized children with acute pancreatitis (18). Earlier this year, Damaan et al and Bineshfar et al described 2 more cases of COVID-19 and pancreatitis (19, 20).

Aslan et al published an interesting case of COVID-19 associated MIS-C, a 12-year-old female, presenting with acute abdominal pain with features of acute appendicitis who developed pancreatitis on the fourth day of admission (21). This may be designated as the first MIS-C-associated pancreatitis in literature. Afterward two more cases of acute pancreatitis associated with a diagnosis of MIS-C were described by Stevens et al (22) and Zubiaurre et al (23). But a cohort of 9 children is so far rare.

A slight male preponderance was seen in most series with MIS-C (9,10) although the reason was not known. A similar observation was seen in this cohort with 6 (66%) males. The age group affected was described as 6 to 12 years by Yosuhara et al (10), but this cohort had 4 years as the youngest child affected similar to Riphagen et al (2).

A median CRP of 112 mg/L was consistent with very high CRP depicted in all the series of MIS-C with or without pancreatitis. Feldstain et al found CRP as an inflammatory parameter was much higher in COVID-19-related MIS-C than seen in acute severe COVID-19 (133 versus 32 mg/L) (9). A low median serum albumin value of 2.6 g/dL was also congruous with the existing data (9,10). Although necrotic pancreatitis was described in one adult case report (24) of COVID-19-associated pancreatitis most cases in pediatric series with or without MIS-C were interstitial pancreatitis as was seen in the present series.

Two cases were found to be COVID-19 RT-PCR positive, other 6 had corona viral antibody (IgG) except one. This is consistent with the findings of Riphagen et al where only 2 (25% in their cohort) were COVID-19 RT-PCR positive (2).

Intravenous gamma globulin is the first step of treatment in these patients, specially with Kawasaki-like or severe shock-like manifestation. So 8(88%) children in our series received gammaglobulin; one could not as she succumbed before procuring the medication. In the series by Ripaghen, this figure was 100% but in the recent meta-analysis, gammaglobulin was administered in a mean of 81% of patients (10).

Various mortality figures were quoted in MIS-C. Feldstein and Yasuhara both depicted a 1.9% mortality, which is much lower than the mortality of 12% among the total MIS-C cases in this study. This figure was higher (22%) when calculated among the group with pancreatitis as both patients who died were in this group. The reason for this higher value might be explained by the lower number of total patients. However, this finding of 2 deaths in the MIS-C group with pancreatitis compared with zero mortality in the fraction of MIS-C without pancreatitis supports the observation of Wang et al (11) who described a more serious illness among COVID-19 patients with pancreatitis. This is the first study to document 3 months follow-up for all survived children with MIS-C and pancreatitis. It showed a complete reversal of pancreatic and cardiac pathology.

The cause of pancreatic injury in COVID-19 is not exact so far bur many explanations have come forward as of now. In a study that explored the distribution of ACE 2 receptors of SARS-CoV-2, it was shown to be more expressed in the pancreas than lung (25). This may explain that pancreas might be a target of SARS-CoV-2. But pancreatitis in MIS-C associated with COVID-19 remains largely unexplained. Whether it is secondary to the cytokine-mediated inflammation or secondary to severe cardiac involvement with reduction of ejection fraction in few cases is still unexplored. As described earlier abdominal symptoms were largely kept nonspecific with pain or diarrhea or vomiting in most of the criteria of MIS-C. A study previously described raised serum lipase without symptoms of acute pancreatitis (26). In our series, all children had raised lipase as well as abdominal pain, which already fulfills the Atlanta criteria (8) for acute pancreatitis. Nevertheless, they (except 2 who died) also had pancreatic imaging (CT/MRCP) to support the diagnosis further. However, further studies are required to explain the mechanisms of pancreatic damage in COVID-19-related MIS-C.

The limitations of this study were small sample size and the retrospective nature of analysis carried out from a single unit. A larger sample size might have altered the findings.

In conclusion, acute pancreatitis can be a part of the organ systems affected by COVID-19-associated MIS-C. Further research involving a larger cohort is needed. But we suggest considering the inclusion of the pancreas as an independent organ involvement in the diagnostic criteria of MIS-C temporally associated with the present pandemic.

## Supplementary Material


